# Cardiac Responses to Alcohol: A Review of Mechanisms and Clinical Implications

**DOI:** 10.31083/RCM47953

**Published:** 2026-05-21

**Authors:** Kristine E Johnston, Aishwarya Kamble, Joanna Lee, Kayla Garley, Vincent M Figueredo

**Affiliations:** ^1^Department of Medicine, St Mary Medical Center, Langhorne, PA 19047, USA; ^2^Division of Cardiology, St Mary Medical Center, Langhorne, PA 19047, USA

**Keywords:** alcohol, heart, cardiac, blood pressure, coronary artery disease, cardiomyopathy, arrhythmia

## Abstract

Alcohol has been appreciated by civilizations for thousands of years. Nonetheless, alcohol has recently been linked to serious cardiovascular diseases. Therefore, this review summarizes the effects of alcohol on the heart based on the most current literature available. This review covers the effects of alcohol on blood pressure (BP), heart rate (HR), autonomic dysfunction, coronary artery disease (CAD), cardiomyopathy, and cardiac arrhythmias. Evidence supports the harmful effects of both acute (binge) and chronic heavy alcohol consumption on BP, HR, autonomic dysfunction, and the risk of CAD, cardiomyopathy, and various arrhythmias. Some studies suggest that mild to moderate drinking may reduce the risk of CAD and arrhythmias, consistent with a potential J-shaped relationship. However, other evidence contradicts this, thereby indicating that alcohol use may increase the risk of developing hypertension, CAD, and atrial fibrillation. At the time of this review, evidence supports the harmful effects of acute and chronic heavy alcohol consumption on the heart. Findings are mixed for mild to moderate drinking and may be influenced by confounding factors, underscoring the need for further research. Given the inconclusive benefits, the authors recommend that no level of alcohol consumption be promoted as beneficial for cardiac health.

## 1. Introduction

The consumption of alcohol dates back through millennia in human history, from beer dating over 10,000 years ago in Israel [1], to it becoming illegal in the USA in the 20th century and now being an accepted part of social and cultural life. On the other hand, the toll that alcohol takes on the human body is far less celebrated, with significant health consequences. Approximately 315,000 deaths worldwide were attributable to alcohol-related injury in 2019 [2]. According to the United States Centers for Disease Control and Prevention (CDC), 178,000 deaths in the United States were related to alcohol between 2020 and 2021, which increased from previous years [3]. Of the 178,000, about two-thirds were related to chronic effects of alcohol, and one-third were related to binge drinking behavior [3]. The preventable deaths related to the use of alcohol and violence or vehicle accidents make alcohol a continued public health concern today.

Standard alcoholic drink servings have been established. One alcoholic drink is equal to 12 ounces of 5% alcohol by volume (ABV) beer, 8 ounces of 7% ABV malt liquor, 5 ounces of 12% ABV wine, or 1.5 ounces of 40% ABV distilled spirit [4]. It is commonly recommended to keep track of the number and type of drinks that one has. This may become complicated by variances in alcohol content, such as beer varieties made with higher ABV than a standard beer, leading to people drinking more than intended and potentially putting them at increased risk of injury.

According to the United States CDC, more than half of adult Americans drink alcohol, 17% binge drink, and 6% chronically drink heavily [5]. Binge drinking is defined as four or more drinks for women, and five or more for men, in one session [5]. Not only does excessive drinking pose health risks, but it also costs the United States economically, with lower work performance, increased needs for criminal justice, and increased costs to the healthcare system [5]. It also has addictive potential, condemning many people to the financial, health, and psychological impacts of chronic abuse.

Alcohol has the potential to affect nearly every body system. It can destroy neurologic cells, contribute to dementia, cause alcoholic neuropathy, and lead to substance use dependency [6]. It can lead to hypertension, cerebrovascular accidents, cardiomyopathy, arrhythmias, and heart disease. Alcohol has teratogenic effects on the fetus [6]. Alcohol has serious gastrointestinal implications on the liver and pancreas, being a significant contributor to alcoholic cirrhosis and pancreatitis [6]. It is associated with cancers of the gastrointestinal tract, as well as breast and ovarian cancers [6]. Alcohol affects the endocrine system by acting on the hypothalamic-pituitary-adrenal axis. Alcohol increases circulating cortisol levels, a hormone used in stress response [7]. While there may be potential benefits of mild to moderate alcohol consumption on insulin sensitivity and reducing the risk of developing type 2 diabetes, excessive drinking is associated with higher risk of type 2 diabetes [7]. There has been debate over the years about whether mild to moderate drinking has potentially beneficial effects on health, especially in preventing cardiovascular disease (CVD) [8, 9]. This review article will explore the effects of alcohol on cardiac health and physiology, describe potential mechanisms of pathology, discuss the most recent evidence available, and provide ideas for future studies to investigate.

## 2. Literature Search Methods

A narrative literature review was conducted to examine the effects of alcohol on the heart and cardiovascular system. Primary literature searches were performed in PubMed to identify peer-reviewed articles published up to December 2025. Articles were selected based on relevance to the review topic, contribution to clinical understanding, and date of publication. Preference was given to recent studies. Reference lists of selected articles were also reviewed to identify further relevant studies. Supplemental targeted searches were performed as needed to capture additional articles not identified through the primary database search. Search terms included combinations of key terms such as “alcohol AND coronary artery disease” or “alcohol AND atrial fibrillation”.

## 3. Alcohol and Blood Pressure, Heart Rate, and Autonomics

The effects of alcohol consumption on the heart can be separated into acute versus chronic effects. Binge drinking or chronic heavy alcohol consumption that leads to blood alcohol content of >0.08% has acute and chronic implications for blood pressure (BP) [10]. Over time, it increases the risk of developing hypertension, and consequently CVD [10, 11]. It is well-known that chronically elevated blood pressures can lead to permanent end-organ damage, with it commonly manifesting as stroke, retinopathy, coronary artery disease, heart failure, renal failure, and aneurysms [11].

Chronic alcohol consumption and binge drinking can lead to arterial endothelial dysfunction [10], a key early step in the formation of atherosclerosis. When the integrity of the endothelium is compromised by a stressor, it becomes pro-inflammatory, more permeable, and has increased oxidative stress [10]. Various amounts of alcohol have been correlated with increased reactive oxygen species (ROS), which are inherently pro-inflammatory and contribute to endothelial dysfunction and atherosclerosis [10]. Nitric oxide, produced by the endothelium, ordinarily enhances vasodilation and prevents platelet aggregation in the arteries; high levels of alcohol are suggested to be inhibitory on nitric oxide, preventing its ability to regulate blood pressure and atherosclerosis [10]. Changes in nitric oxide are pivotal in the process of endothelial dysfunction, contributing to hypertension and end-organ damage [11, 12]. Conversely, chronic alcohol use is thought to increase circulating levels of endothelin-1 and angiotensin II, both of which are vasoconstrictive compounds that contribute to elevated blood pressures [10]. On the other hand, the effects of mild to moderate drinking on BP have been widely debated, with older studies suggesting protective effects on vasculature and cardiac outcomes [8, 10]. It has previously been established that endothelial dysfunction leads to increased permeability endovascularly, allowing low-density lipoprotein (LDL) to form fatty streaks in the intima layer in early plaque formation and atherosclerosis [13]. Endothelial dysfunction leads to plaque formation and reduced compliance of vessels, further increasing vascular resistance, and ultimately contributing to hypertension and end-organ damage [11, 12].

The effect of chronic alcohol consumption on the sympathetic system plays a role in the development of increased BP and CVD [14, 15]. In addition, alcohol drinkers have more resistant hypertension than their non-drinking counterparts. A recent trial continues to support this, with non-drinking control groups responding better to standard drug therapies when compared with drinking groups [16]. On the other hand, the effects of alcohol on BP and sympathetic tone are more nuanced when measuring its effects immediately following drinking versus chronically over years. Trials that investigate the acute effects of alcohol have suggested that evening binge drinking leads to increased sympathetic tone the morning after [14, 17]. Paradoxically, it acutely increases heart rate (HR) and reduces vascular resistance and BP during orthostatic challenge, making syncope and exaggerated physiologic responses to other antihypertensive medications more likely [18, 19]. One trial found delayed muscle sympathetic nerve activity immediately after drinking, which ordinarily helps normalize BP when encountering orthostatic situations [18]. Another trial found that the BP-lowering effects immediately following alcohol consumption lasted for hours and made the effects of alpha-1 blockers more pronounced [19]. A trial from 2021 found that binge drinking acutely lowered vagal tone (correlated with heart rate variability) during sleep, and increased sympathetic activity, contributing to alcohol-associated pathology [20].

Another trial published in 2025 found that binge drinking led to increased HR, aortic pulsatile load, and aortic wave reflection, which can contribute to long-term effects such as hypertension, end-organ damage, and CVD [21]. This was a trial that included 33 men and women averaging age 25 years, body mass index (BMI) 27 kg/m^2^, and resting systolic BP less than 120 mmHg as baseline characteristics. Chronic drinking has been observed to lead to elevated BP at night [22]. One randomized controlled trial attempted antihypertensive treatment in alcohol groups with chronically elevated nocturnal BP and found that standard antihypertensive treatments were not as effective in these groups compared with non-drinking groups [22]. Overall, the evidence suggests an initial decrease in BP immediately following alcohol consumption, followed by increased BP hours later [14, 17].

While there is clear evidence of increased BP with chronic consumption of alcohol over time, there is also evidence of autonomic dysfunction in heavy alcohol drinkers [23], potentially contributing to orthostatic hypotension and syncope. This is not surprising, as alcohol is known to have effects on the nervous system centrally and peripherally [23]. Possible mechanisms to explain orthostatic changes include alcohol-associated neuropathy and disruption of baroreflex function [20, 23]. Baroreflex regulation is important in maintaining BP homeostasis. A recent systematic review found evidence of abnormalities in cardiovascular reflexes, among other symptoms of peripheral neuropathy, such as erectile dysfunction, in chronic alcohol users [23]. A separate randomized crossover study found dysfunction of cardiac vagal tone and baroreceptor function during sleep [20]. All in all, the autonomic nervous system plays a crucial role in regulating BP and can be adversely affected by excessive drinking.

There is potential for novel modalities of treatment for patients with alcohol use disorder. Recent evidence suggests that pregnenolone, which can mediate sympathetic responses, may play a therapeutic role on a physiologic level for patients with alcohol use disorder [15]. Chronic alcohol use has been associated with a loss of Gamma-Aminobutyric Acid (GABA) receptor-mediated inhibition [24]. Typical GABA function helps to mediate mood, sleep, and seizures. Allopregnenolone has been suggested to enhance GABA function by increasing production of GABA subunits, combating the effects of alcohol dependence [24]. It has also been shown to have antidepressant effects in animal models, and antiepileptic effects in alcohol-dependent animals [24]. One experiment on rats found that supplemental GABA reduced blood pressure [25]. Perhaps exogenously administered pregnenolone could have similar effects. However, further studies are needed to investigate benefits and adverse effects, if any, of these types of steroid infusions.

**Fig. 1.**
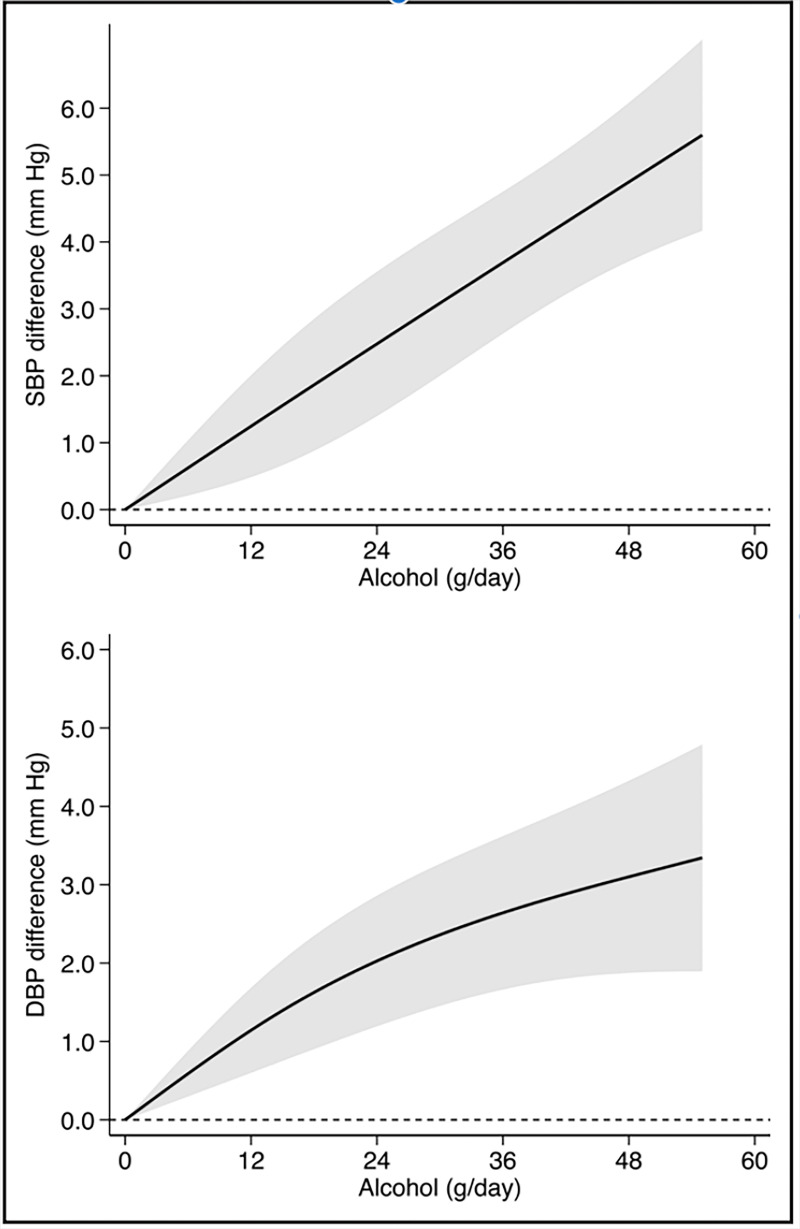
**Alcohol dose-dependent changes in systolic blood pressure (SBP) and diastolic blood pressure (DBP) across cohort studies in meta-analysis**. Reproduced with permission by Wolters Kluwer Health, Inc [26].

In a systematic review from 2023, a linear increase in systolic BP was correlated with increased grams of alcohol consumed per day [26]. This trend is visualized in Fig. 1 (Ref. [26]). In a separate systematic review, it was found that consuming 24 grams of alcohol per day was a cutoff marker for increased risk for hypertension [27]. As a reference, a 1.5 oz serving of 80-proof liquor has approximately 14 grams of alcohol, while a 16 oz serving of beer with 7% alcohol would have approximately 26 grams [28]. The debate with regards to potentially beneficial — or at least not harmful — effects of mild and moderate drinking still merits further research.

Another mechanism by which alcohol can contribute to increased risk of hypertension is by alcohol-associated weight gain. Alcohol can contribute to weight gain by increased caloric intake. Additionally, it is theorized that weight loss can lower systolic BP in individuals with hypertension [29]. Thus, reducing alcohol intake would be expected to contribute to weight loss and lowered BP in chronic drinkers. One study correlated a reduction of alcohol intake with lowered systolic BP and weight loss after only six weeks of reducing alcohol intake by 80% [30]. Regardless of mechanism, a clear association can be found between alcohol and hypertension.

### 3.1 Alcohol and Coronary Artery Disease

**Fig. 2.**
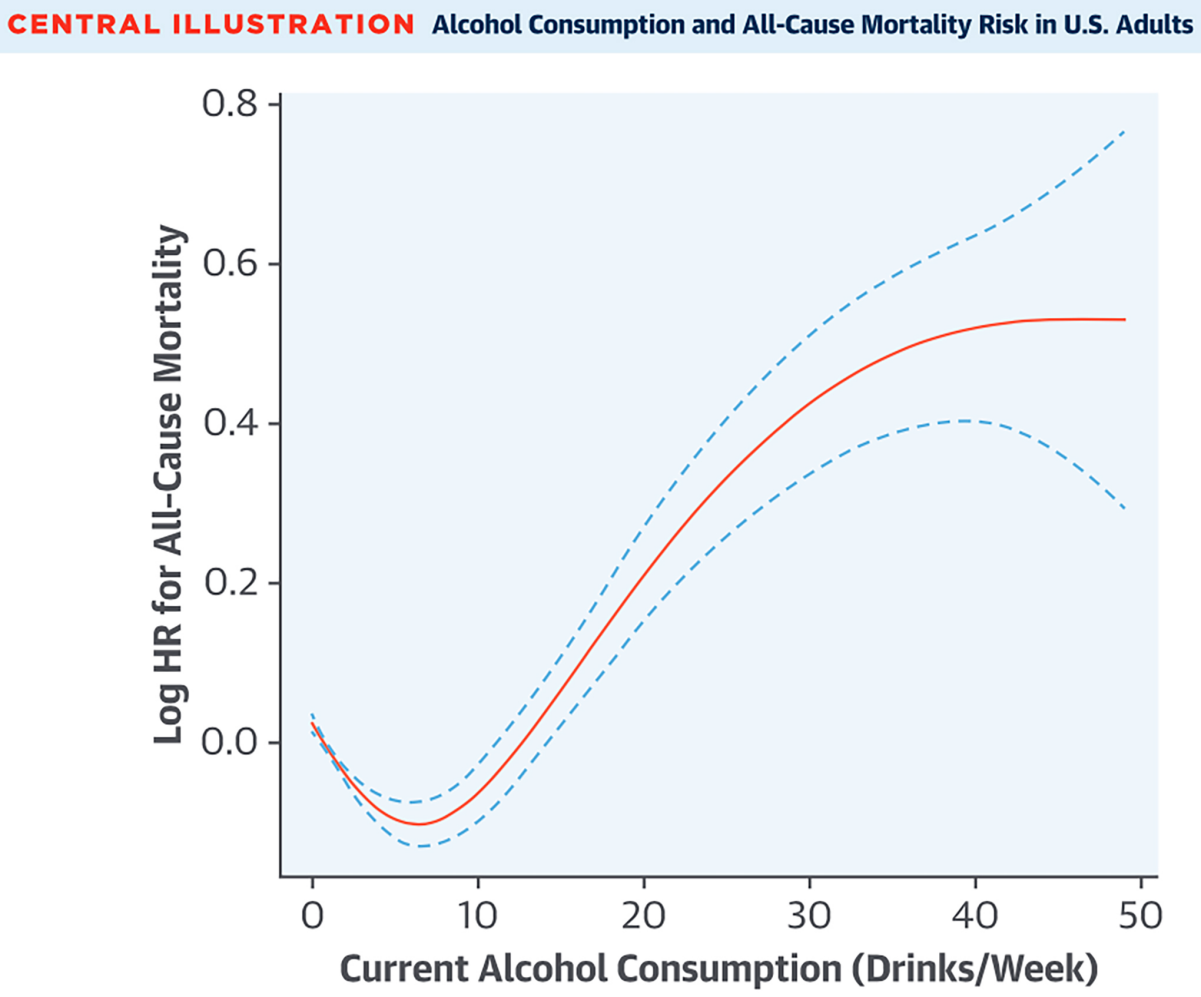
**Alcohol consumption and all-cause mortality in the United States portrayed as a J-shaped curve, suggesting potential benefits with low alcohol consumption**. Reproduced with permission by Elsevier [9].

Coronary artery disease (CAD) is the leading cause of death worldwide [31, 32]. There are many factors that can contribute to this diagnosis, with alcohol being a potential mediator. There has been controversy surrounding this association as the relationship between alcohol and CAD is complex, confounded, and dose-dependent. For many years, studies have attempted to demonstrate a J-shaped relationship between alcohol consumption and CAD, and previously suggested that light to moderate drinkers (1–2 drinks per day) had lower CAD risk when compared to those without consumption at all [8, 9]. Fig. 2 (Ref. [9]) visualizes a J-shaped curve that correlates alcohol consumption with all-cause mortality, where CAD is a recognized cause of mortality. Notably, because this curve is not specific to CAD, it cannot be concluded that light alcohol consumption is protective against CAD. In contrast, heavy drinkers previously showed higher risk for the development of CAD [33, 34]. More recent studies, such as meta-analyses and mendelian randomization studies, are now challenging this J-shaped relationship [35, 36, 37].

Further complicating this controversy, no randomized clinical trials exist regarding this association due to ethical implications of inducing CAD in participants by asking them to drink alcohol. In fact, one trial that was looking at modest alcohol consumption, led by the National Institutes of Health, was discontinued because of ethical concerns [38]. Hence, the unfeasibility of these types of studies leaves this question unanswered. Although there are no randomized clinical trials that directly conclude how much alcohol is needed to cause ischemic cardiac disease, there have been numerous observational studies with various techniques that aim to assess this correlation. There have also been studies done to observe the immediate effects of binge drinking on various physiologic parameters (see section on BP) that could potentially lead to chronic diseases and eventually CAD down the line, if left unchecked.

A recent article published in 2022 sought to determine the risk of CVD associated with different amounts of habitual alcohol consumption [35]. The authors reanalyzed how lifestyle and behavioral factors influence the known J-shaped link between alcohol consumption and CAD. They then used traditional mendelian randomization (MR) and non-linear MR (NLMR) as part of a cohort study to explore how variations in genetics might affect different levels of alcohol intake and risk of developing CAD [35]. They removed specific single-nucleotide variants associated with risk factors for CAD to reduce the probability of pleiotropy. It included 371,463 participants who consumed an average of 9 drinks per week (46% of the participants were male). The study found that even light to moderate alcohol consumption may increase the risk of CAD, challenging the theory that alcohol has some cardioprotective effects. The alleged benefits may be attributed to healthier lifestyles among moderate drinkers, though this would require separate analysis. Genetic evidence may show a causal, dose-dependent rise in risks, suggesting that any reduction in alcohol intake could improve heart health in certain populations [35]. The study may still be limited by unforeseen pleiotropy not accounted for in their selection.

In contrast, a systematic review and meta-analysis published in 2011 evaluated the effect of alcohol intake on various cardiovascular outcomes [8]. They included 84 prospective cohort studies with close to one million participants and greater than 94,000 cardiovascular events to determine the relation between alcohol intake and cardiovascular events. The outcome of this systematic review and meta-analysis showed that moderate alcohol intake was associated with a lower risk of several cardiovascular events. In addition, the analysis also showed a dose-response pattern, in which those who consumed around one to two drinks per day had the lowest risk of CAD, but those who drank heavier did not see these benefits and may have increased health risks. While this analysis did suggest the traditional J-shaped relationship between alcohol intake and CAD, the researchers cautioned that these results are from observational data, which are open to confounding variables including, but not limited to, lifestyle and diet [8]. Another study from 2014 stratified demographics in cases of first myocardial infarction across 52 countries and found an association with moderate alcohol consumption and reduced risk of infarction compared with nondrinkers, but this finding was not consistent across geographical regions [39]. More research is needed to delineate patterns across age groups, geographical regions, sexes, and genetics.

One could conclude that studies show a protective factor of CAD with low to moderate alcohol ingestion. However, it is important to understand that many of the studies are observational studies, which will inevitably contain confounding variables. The current evidence shows a more unified conclusion regarding binge drinking and chronic heavy alcohol drinkers, who are at higher risk for CAD. The public should be made aware that mild to moderate drinkers may still be at risk as current data available shows divided conclusions.

The discussion surrounding the J-shaped curve and its relationship with alcohol consumption and long-lasting health effects is more recently debated [35, 36, 37]. As previously mentioned, light to moderate drinkers may inherently lead healthier lifestyles that could be causing the initial part of the J-curve [35, 37]. Another potential bias could be from abstainers, who may choose to abstain to accommodate, or make up, for worse overall health and lifestyles [37]. Mendelian studies have recently suggested that even modest amounts of alcohol consumption do not provide evidence of cardiac protection, and that cofounding lifestyles/factors may have biased previous results [35]. The biobanks used for genetic studies are also largely from white populations, limiting the generalizability of the J-curve. A recent meta-analysis from 2023 selected 107 studies after attempting to remove confounders [37]. They found no protective benefit from occasional to low-volume drinking on all-cause mortality, and an increase in all-cause mortality in those who drink more than 25 g per day. They also found that women are at higher risk of all-cause mortality when drinking more than 25 g per day [37]. It is likely in the interest of the scientific world to reassess the validity and generalizability of the J-curve, and to reassess how any potential benefits of alcohol are marketed to the masses.

Potential mechanisms by which significant alcohol consumption can lead to CAD were previously described in the section on BP. Heavy drinking can lead to endothelial dysfunction, inflammation, and ultimately plaque formation in coronary arteries [10]. Additionally, the effect of alcohol on blood cholesterol is complex and is dependent on the amount of alcohol consumed and likely genetic predisposition to dyslipidemia as well. Previous studies have consistently suggested an increase in high-density lipoprotein (HDL) — also known as good cholesterol — with mild to moderate levels of alcohol consumption [40, 41], however these studies are not without limitations. On the other hand, heavy drinking is associated with higher levels of triglycerides [40] — commonly attributed to atherosclerosis — as well as damage to the liver, which processes cholesterol in the human body.

### 3.2 Alcohol and Cardiomyopathy

Alcohol induced cardiomyopathy (ACM) is an acquired form of dilated cardiomyopathy (DCM) resulting from prolonged and excessive alcohol intake. Generally, over 80 g per day over a span of 5 years or more has been associated with ACM [42]. In older population-based studies, modest-to-moderate alcohol consumption has been associated with favorable effects on the cardiovascular system, including ischemic heart disease [8]. Furthermore, an older study previously reported a lower incidence of heart failure with moderate alcohol consumption compared to patients who drank less than one drink per week [43]. However, excessive alcohol consumption has been associated with decreased myocardial contractility [44]. Potential mechanisms include high oxidative stress and apoptosis, which can lead to cardiomyopathy and clinical heart failure [44].

Alcohol is cited as a leading cause of dilated non-ischemic cardiomyopathy by the American Heart Association [45]. The prevalence of ACM among patients with unexplained DCM has been reported in a range of 3% to 40% [46, 47, 48]. Furthermore, ACM-associated mortality in the US has increased from 2010 to 2020 [46]. On the other hand, data from 2012 to 2020 in Europe showed that the ACM-attributed mortality rate has declined in the past decade, paralleling a reduction in alcohol consumption [49]. Women tend to drink less alcohol than men [50] which, in part, explains why women may be under-represented in studies related to ACM. In 2019, one study estimated that the prevalence of 1 in 1471 hospitalizations were related to ACM, with a ratio of 8:1 between men and women [51].

One of the largest studies linking alcohol use and cardiac remodeling is from South Korea, including almost 50,000 participants. The study classified patients into different groups based on daily alcohol consumption and evaluated echocardiographic changes. Very heavy drinkers (more than 60 g of consumption per day) had significantly increased ventricular wall thickness and impaired diastolic function of the left ventricle compared to non-drinkers [52]. Furthermore, population-based cohort studies such as HUNT (Trøndelag Health Study) suggested that light-to-moderate-alcohol intake was not associated with cardiac remodeling [53], while the CARDIA (coronary artery risk development in young adults) study showed no adverse remodeling for patients who predominantly consumed wine [54].

The pathogenesis of ACM is not well understood, but likely involves a combination of direct toxic effects of alcohol and genetic predisposition [55]. Direct toxic effects of alcohol have been studied in both acute and chronic intake. Acute intake in binge drinking has been associated with a transient increase in troponins and myocardium-skeletal muscle ratio seen in T2-intensity signaling MR imaging within 24 hours after consumption [56]. However, these findings were reversed in repeat studies a week later. No late gadolinium enhancement was found to suggest scar tissue, suggesting reversible changes in isolated binge drinking [56].

**Fig. 3.**
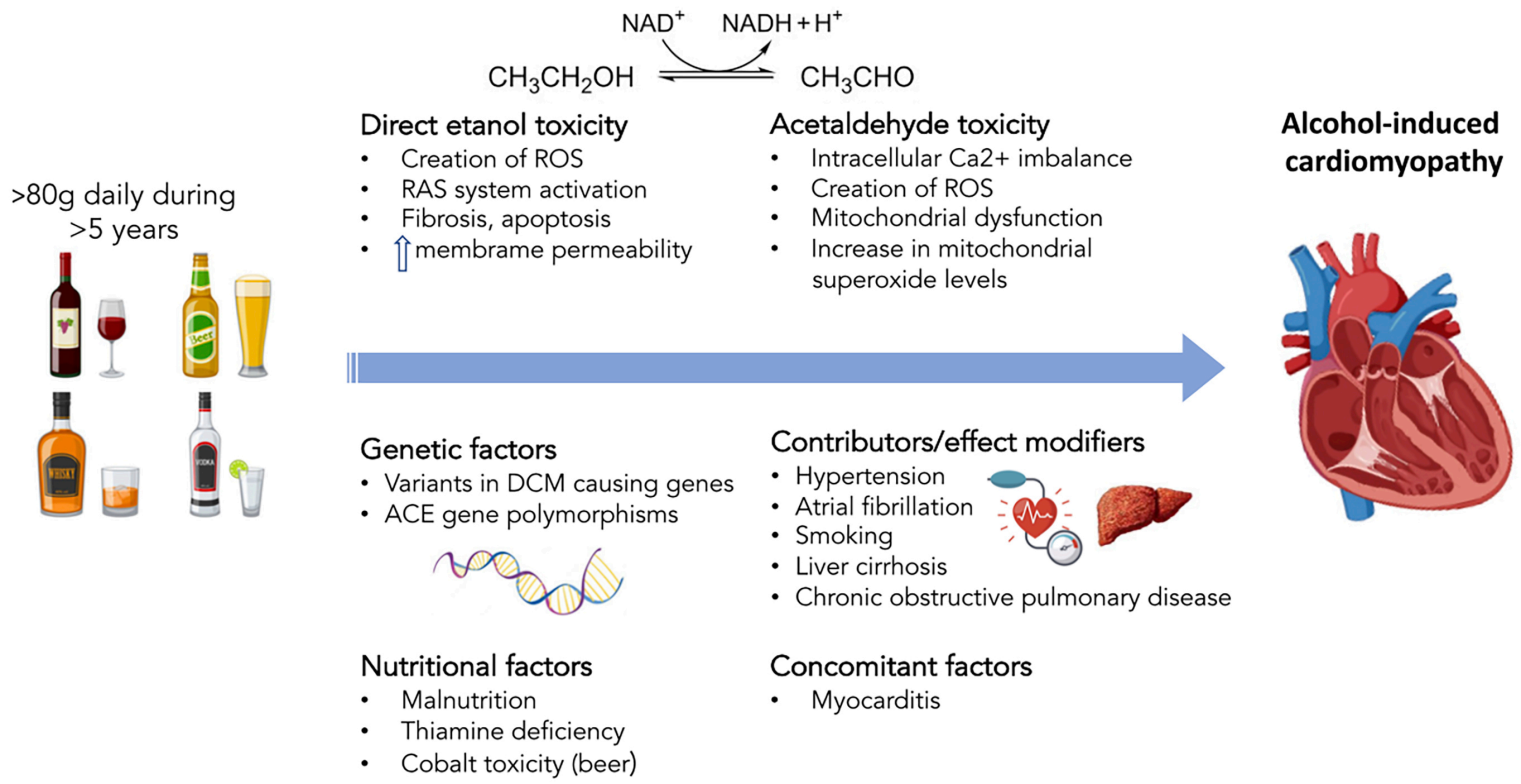
**Pathogenic mechanisms of alcohol-induced cardiomyopathy**. ACE, angiotensin-converting enzyme; DCM, dilated cardiomyopathy; RAS, renin–angiotensin system; ROS, reactive oxygen species. Reproduced with permission by Oxford University Press [42].

Chronic alcohol intake, however, has direct toxic effects to the cardiac myocytes that lead to apoptosis, fibrosis, and impaired contractility of the ventricles in animal models and humans [57]. Alcohol consumption leads to production of acetaldehyde, produced by alcohol dehydrogenase in the liver [58]. Acetaldehyde acts on the sarcoplasmic reticulum by reducing the supply of calcium and impairing the cellular excitation-contraction between actin and myosin [59, 60]. Acetaldehyde also contributes to mitochondrial dysfunction by generating ROS, further producing oxidative stress and leading to changes in the mitochondrial oxidative phosphorylation system [61]. This pathology is demonstrated in Fig. 3 (Ref. [42]).

Over 100 genes have been implicated in the risk of developing DCM, with about 40 of them more strongly correlated Harakalova *et al*. [62]. Far fewer have been identified specifically to ACM. Recent studies have suggested prevalence of variants in DCM-causing genes, known as titin-truncating variants (TTNtv) [63]. Titin (*TTN*) is an important sarcomere protein that provides elasticity to cardiac muscle [64]. TTNtv continues to be reported as the most common mutated protein found in genetic DCM in recent literature [64, 65]. The theory previously gained support as the study showed ACM patients with TTNtv had 8.7% absolute reduction in LVEF compared to those without TTNtv, suggesting a double-hit hypothesis of ACM caused by combination of genetics and environmental factors [63]. A retrospective observational study, published in 2025, investigated over 3000 subjects in families with TTNtv related DCM for additional factors leading to clinical disease [65]. Various stressors, such as pregnancy, specific chemotherapies, and alcohol were suspected to be environmental factors increasing risk in genetically susceptible people [65]. The strongest associations of developing DCM were in younger populations, with young males being especially affected by excess alcohol intake [65]. The same study found that early initiation of beta receptor or renin-angiotensin system-blocking agents before disease onset could be beneficial in high-risk groups, but noted that further studies would need to be done before a formal recommendation is made [65]. An older study of 57 participants found that angiotensin-converting enzyme (*ACE*) gene polymorphism was reported to be weakly associated with ACM [66]. The study compared *ACE* genotypes of ACM patients with those without signs of cardiomyopathy [66]. They found that 57% of ACM patients had the deletion-deletion (DD) *ACE* polymorphism, which is linked with higher levels of *ACE* and associated with cardiovascular issues, vs 7% in the non-ACM group [66]. Various genes have been associated with DCM but not necessarily with ACM yet. However, this does not mean that they are not related. Further research is needed to untangle the relationships between multiple genetic mutations and their combined risk. For example, various mutations have been implicated in the involvement of Brugada and long QT syndromes, and the development of arrhythmias [64].

Treatment of ACM includes a combination of lifestyle modifications, pharmacological treatment, and supportive care. Observational studies have addressed this issue and demonstrated improvement of left ventricle (LV) function with alcohol abstinence from a period between 10 weeks to 1.5 years [67, 68, 69]. A more recent study in 2018 that followed 101 patients with ACM showed that persistent heavy alcohol intake did not result in left ventricular ejection fraction (LVEF) recovery in the span of 82 months, while 42% of the patients who reduced intake or abstained had improvement in LVEF [70]. Among the 42%, there was no difference in recovery rate between those who abstained and those who continued to drink at low-moderate levels [70]. Older studies from 2002 to 2015 observed similar findings, wherein complete abstinence from alcohol did not have additional LVEF recovery compared to those who controlled their alcohol intake to less than 80 g per day [71, 72]. A study from 2015 that compared 94 patients with ACM to 188 patients with idiopathic DCM found that the ACM group showed significant improvements in cardiac function compared with the idiopathic group over time, signifying some reversibility that may have involved reduced alcohol intake [72]. Other lifestyle modifications recommended to patients with generalized HF are a diet consisting of fruits, vegetables, and whole grains, a low-sodium diet, and fluid restriction [73].

**Fig. 4.**
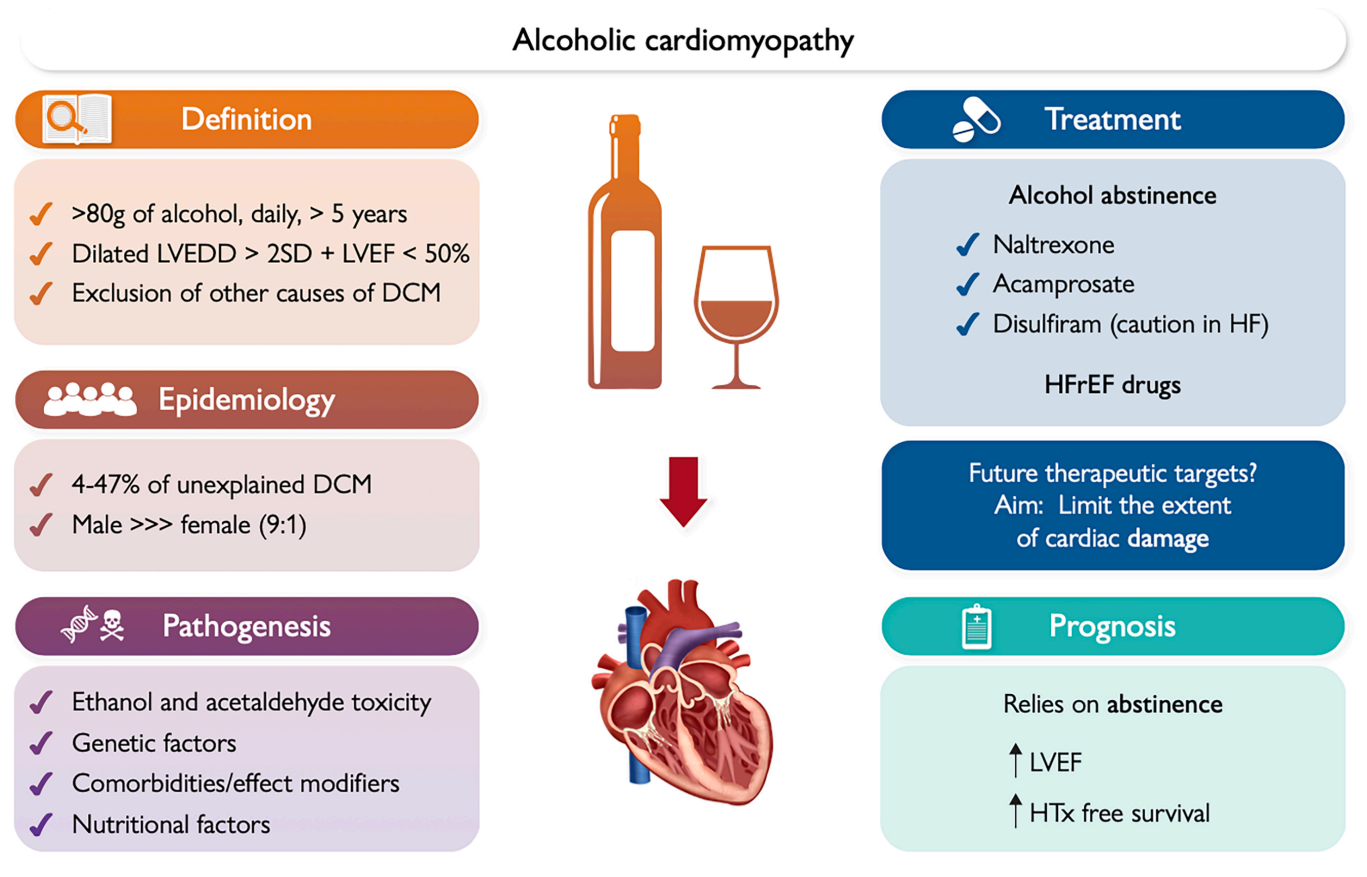
**Clinical overview, pathogenesis, treatment, and prognosis of alcoholic cardiomyopathy**. DCM, dilated cardiomyopathy; HF, heart failure; HFrEF, heart failure with reduced ejection fraction; HTx, heart transplant; LVEDD, left ventricular end-diastolic diameter; LVEF, left ventricular ejection fraction; SD, standard deviation. Black arrows mean that the favorable prognosis of alcohol-induced dilated cardiomyopathy may rely on abstinence from alcohol, increasing the chances of improved LVEF over time and heart transplant survival. Reproduced with permission by Oxford University Press [42].

Pharmacological treatment options include goal-directed medical therapy (GDMT) for heart failure with reduced ejection fraction (HFrEF), consisting of angiotensin receptor–neprilysin inhibitor, β-blockers, sodium–glucose cotransporter 2 inhibitors and mineralocorticoid receptor antagonists [74]. In patients with end-stage disease of LVEF of less than 15%, cardiac transplantation may be the only option for those who achieve complete abstinence. In the 2015 study involving 94 patients with ACM, 15% underwent cardiac transplantation [72]. Other pharmacological treatment options for alcohol use disorders have been recommended for ACM patients, such as the opioid antagonist naltrexone and the non-competitive N-methyl-D-aspartate (NMDA) receptor blocker acamprosate. Disulfiram has previously been considered as well. However, disulfiram-alcohol reaction poses dangers such as inducing electrocardiogram (EKG) changes and enhancing chest tightness and dizziness [75]. There have been reported cases of deaths caused by disulfiram in patients with underlying ACS and HF, making it contraindicated in these groups and disqualifying many patients with ACM from using disulfiram for deterrence therapy [76]. On the other hand, naltrexone and acamprosate do not present cardiac-related complications or contraindications, and have shown deterrence from alcohol consumption when used in combination with psychological therapies [75]. A visual summary of alcoholic cardiomyopathy is provided in Fig. 4 (Ref. [42]).

### 3.3 Alcohol and Atrial Arrhythmias

Atrial fibrillation (AF) is the most common sustained arrhythmia, projected to affect over 12 million Americans by 2050 [77], and greater alcohol intake predicting incidental AF [78]. Patients with a history of symptomatic, paroxysmal atrial fibrillation reported alcohol as the number one trigger of their symptoms [79]. A randomized trial from 2020 involving 140 participants demonstrated that abstinence from alcohol significantly reduced AF burden [80], emphasizing the importance of lifestyle modification on cardiac health.

The relationship between alcohol and AF is nuanced when comparing mild to moderate consumption with excessive consumption. Prior studies suggested low to moderate drinking was not significantly associated with developing atrial fibrillation [81, 82, 83]. However, a study in 2014 suggested otherwise [84]. That study followed over 79,000 people who had no known history of atrial fibrillation, and found that even modest levels of alcohol consumption increased the risk of developing atrial fibrillation [84]. What is more supported in the literature is that excessive alcohol intake contributes to the development of arrhythmias [81, 85]. Binge drinking may precipitate a phenomenon known as “holiday heart syndrome”, wherein patients develop cardiac arrhythmias after episodes of excessive drinking [85]. Holiday heart syndrome earned its name due to heavy alcohol consumption around holidays and commonly presents as AF [85], though other supraventricular arrhythmias may also occur [81, 86, 87].

**Fig. 5.**
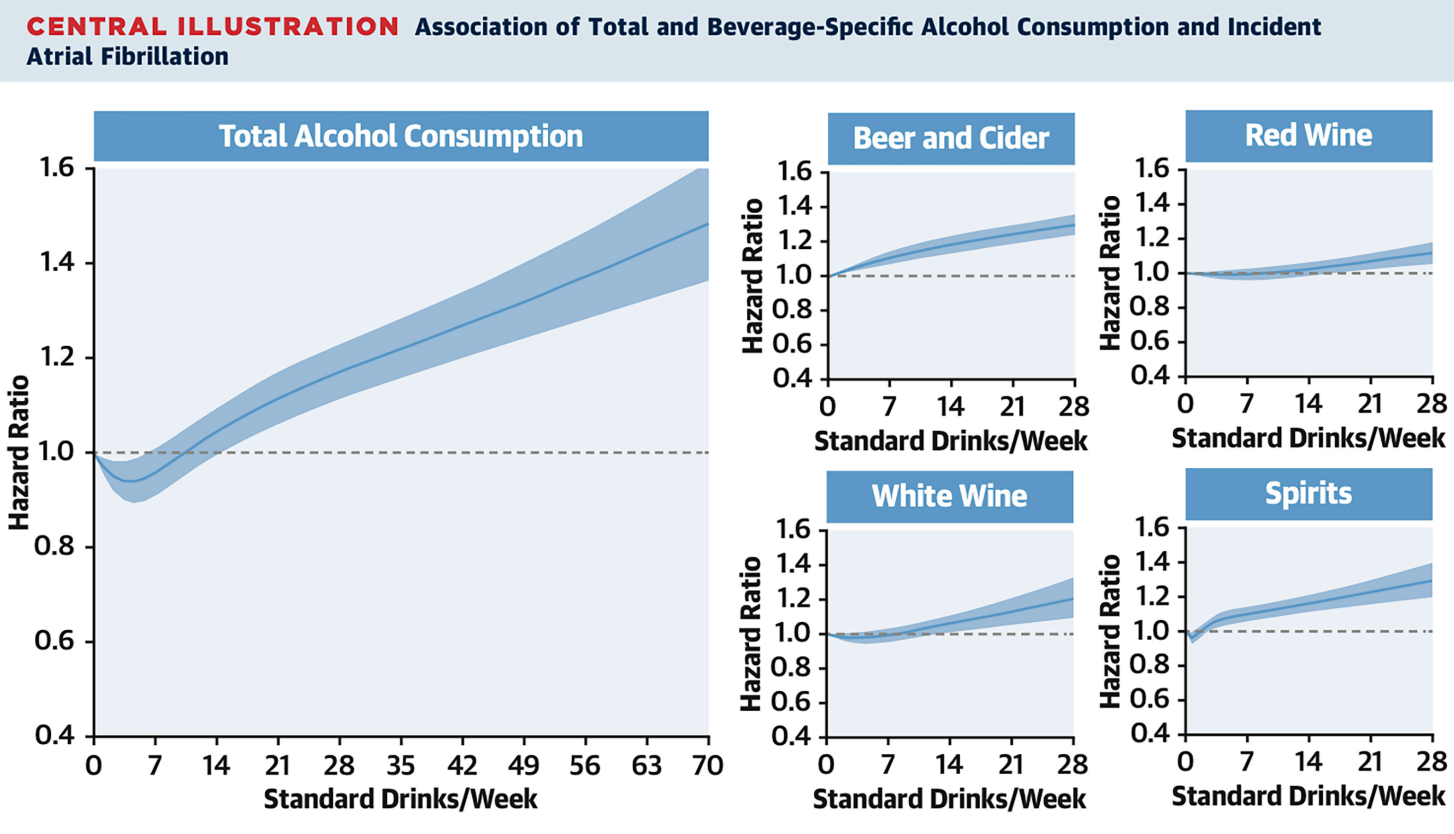
**Association of total alcohol intake with atrial fibrillation (AF)**. AF shows distinct risk trajectory. Reproduced with permission by Elsevier [88].

The United Kingdom (UK) Biobank is a valuable resource comprising biographical, genetic, and health data from a cohort of 500,000 adults. It is designed to provide insights into the causes and risk factors of a wide range of human diseases. Several studies have analyzed the population used in the Biobank to study the association of alcohol with arrhythmias. One study from 2021 of over 400,000 individuals found that low alcohol intake, defined as less than 56 g of alcohol per week, was associated with a low risk for developing AF [88]. Interestingly, when looking at specific types of alcoholic beverages, consumption of any amount of beer or cider was associated with an increased risk for AF, as seen in Fig. 5 [88]. These findings suggest that both the type and amount of alcohol may influence AF risk. In contrast to this study, a meta-analysis conducted in 2014 found that even moderate alcohol consumption was associated with increased risk of atrial fibrillation [84]. Binge drinking and chronic moderate-to-high intake are recognized AF risk factors [81, 83, 85]. The association between alcohol and AF does not clearly exhibit a J-shaped curve [89]. A meta-analysis suggested a J-shaped curve for women, but a linear relationship for men [89]. This suggests that (1) there may be no safe amount of alcohol for men, and (2) women may inherently have more cardioprotective physiology during alcohol consumption, until meeting a threshold after which AF risk increases. More research is needed to differentiate between sexes for the effect of alcohol on AF.

The genetic predisposition to atrial fibrillation is complex. A study from 2020 that used the UK Biobank assessed a polygenic risk score (PRS) specifically to examine how genetics might play a role in developing AF [90]. This study suggested that genetic predisposition may play a role in risk of developing AF, and could help determine whether a person chooses to consume or avoid alcohol [90]. The diverse results suggest that the association between alcohol and the risk of developing AF is nuanced and multifactorial. A prospective cohort study published in 2024 stratified 400,000 individuals for risk of developing atrial fibrillation based on genetic predisposition and physical frailty [91]. The study correlated risk with 142 genetic polymorphisms associated with atrial fibrillation and found that high genetic and frailty profiles were associated with significant risk of developing atrial fibrillation [91]. Sodium Voltage-Gated Channel Alpha Subunit 5 (*SCN5A*), a gene that encodes a part of the cardiac sodium channel, has been correlated with arrhythmias, Brugada syndrome, and long QT syndromes [64]. A study on mice published in 2023 demonstrated gain-of-function of the sodium channel with mutation to *SCN5A*, suggesting enhanced atrial excitability and potential for atrial arrhythmias [92]. Another Biobank study published in 2024 found significant genetic associations between atrial fibrillation and mutations in *TTN*, ribosomal protein L3-like (*RPL3L*), plakophilin 2 (*PKP2*), catenin α3 (*CTNNA3*), cardiac-enriched FHL2-interacting protein (*C10orf71*), and lysine demethylase 5B (*KDM5B*) [93]. Several of these genes play roles in cardiac structure that, when mutated, increase risk of cardiomyopathy and arrhythmias, suggesting that one disease process naturally could promote another. Further studies could further investigate the intricate relationships between several cardiac disease processes and how external factors affect the development of atrial arrhythmias.

**Fig. 6.**
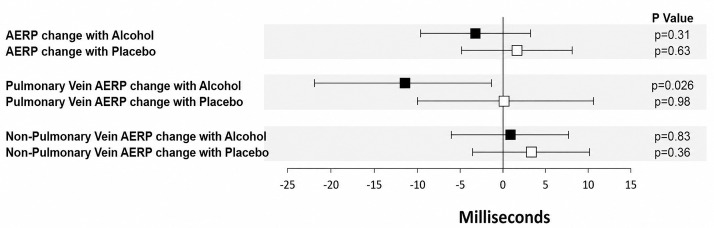
**Change in AERPs during alcohol and placebo infusions blue squares denote changes in AERP with the alcohol infusion, and red squares denote changes with placebo**. Error bars represent 95% confidence intervals. Alcohol significantly shortened AERP compared with placebo. Reproduced with permission by Elsevier [87]. AERP, atrial effective refractory periods.

Reduced atrial effective refractory periods (AERP) are known to be a risk factor to developing AF because they increase the risk of electrical impulses re-entering the atria [94] and contribute to circulating electrical loops. Several studies suggest that alcohol decreases AERPs and conduction velocity, predisposing to AF [87, 95]. AERPs measured around the pulmonary veins were reduced in participants brought to a blood alcohol level of 0.08% blood alcohol concentration (BAC) compared with controls [87]. This study investigated a rather high BAC and may not be representative of low BAC, but it does paint a picture of how binge drinking or heavy chronic drinkers may precipitate paroxysmal AF. A forest plot from this study is provided in Fig. 6 (Ref. [87]).

Premature atrial contractions (PACs) are often benign, yet frequent PACs predict AF, stroke, heart failure, and death [96]. Despite the previously described evidence suggesting that alcohol intake is associated with AF, there is less evidence to support that alcohol consumption contributes significantly to the development of PACs. Two cohort studies did not find that alcohol was associated with increased PACs [97, 98]. Further, the MunichBREW Study, consisting of Oktoberfest participants, did not find a relationship between acute alcohol intoxication and PACs, but did find a positive relationship for other arrhythmias, primarily sinus tachycardia [99]. Conversely, a Japanese study linked moderate to heavy consumption with increased PACs and AF [100]. To date, an association between alcohol and PACs remains unclear.

Studies prior to 2020 examined the association of alcohol consumption with different types of supraventricular arrhythmias, other than AF. In one UK Biobank analysis, neither daily nor occasional alcohol use (1–4 times/week) predicted supraventricular tachycardia (SVT) hospitalizations [101]. Patients with SVT were also less likely than those with AF to cite alcohol as an arrhythmia trigger in another study, but did report that beer was a more common trigger compared with other alcohols [102]. The MunichBREW II study, published in 2024, found increased sympathetic activity associated with atrial tachycardias and PACs in binge drinking in a young and healthy population [103]. Several participants experienced atrial fibrillation and ventricular arrhythmias as well. The uniqueness of this study, however, also lies in the fact that they were able to correlate different arrhythmias during different phases of the drinking event due to their monitoring strategy. They found that atrial tachycardias increased as BAC increased, but PACs were more common during recovery phase [103]. Collectively, current data is starting to emerge for correlations between alcohol and other SVT, and should be further studied.

### 3.4 Alcohol and Ventricular Arrhythmias

**Fig. 7.**
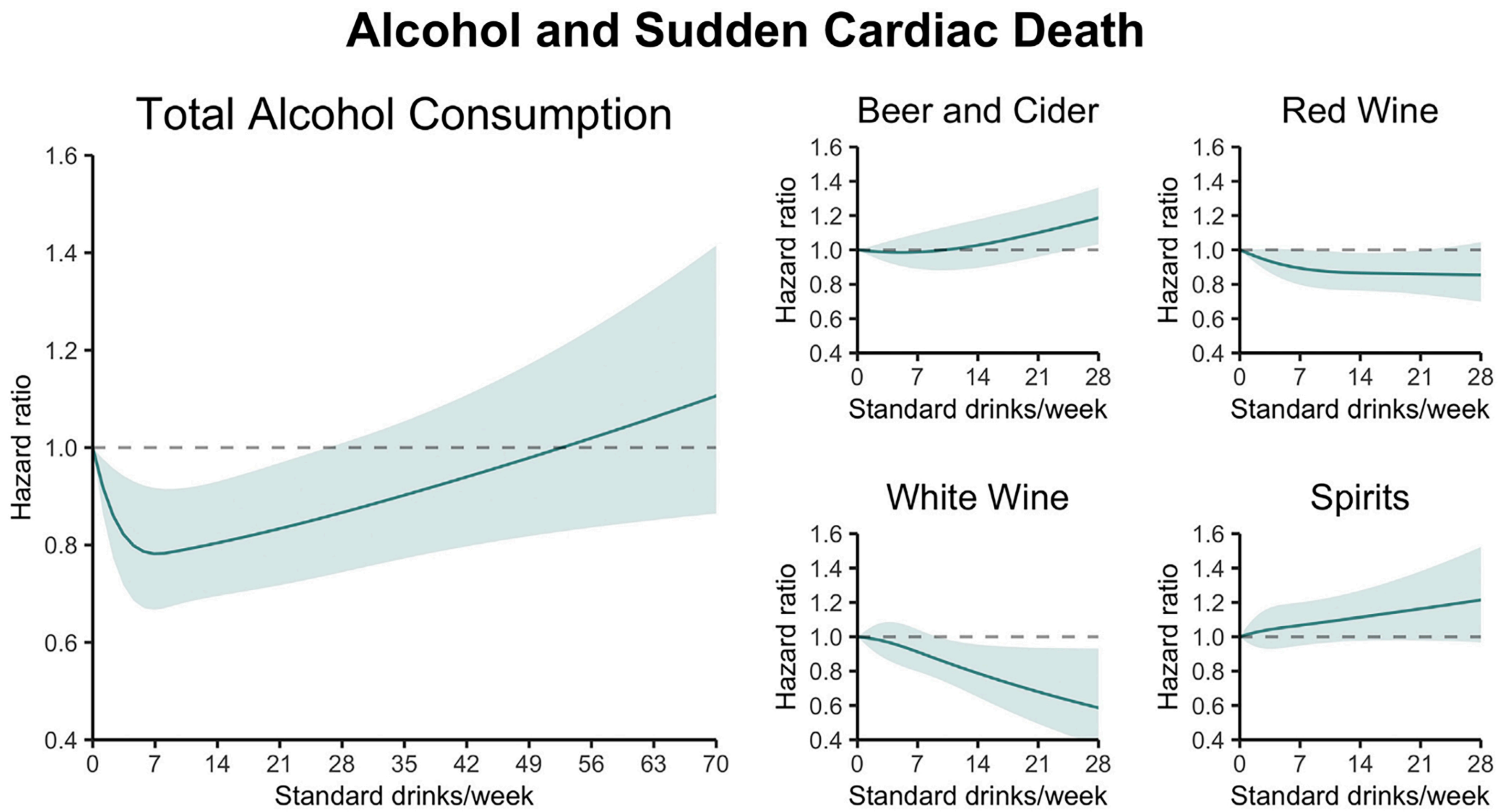
**Association of total alcohol intake with sudden cardiac death (SCD)**. The figure depicts a U-shaped association between alcohol consumption and SCD. Reproduced with permission by Elsevier [104].

A study from 2022 using UK Biobank data showed a U-shaped curve between alcohol intake and risk of sudden cardiac death (SCD), though no significant relationship was observed for outcomes in ventricular tachycardia (VT) or ventricular fibrillation (VF) [104]. This U-shape is visualized in Fig. 7 (Ref. [104]). Conversely, another Biobank study from 2024 differentiated between mild, moderate, and heavy drinkers and found no clear association between alcohol consumption and risk of SCD or ventricular arrhythmias [105]. In an older study of heavy drinkers who presented with ST elevation myocardial infarction (MI) requiring percutaneous coronary intervention (PCI) had a higher odds ratio of developing VF prior to PCI procedure [106]. Among patients with cardiomyopathy, alcohol abstinence was linked to fewer arrhythmic events, including episodes of sustained VT/VF and sudden death [107]. Another study reported no clear association with mild to moderate consumption [108]. Further research is needed to determine if any amount of drinking increases the risk of ventricular arrhythmias.

Analyses from large cohort studies over the past three decades suggest that consuming around one drink per day may correlate with the lowest SCD risk even when compared with non-drinkers [104, 109, 110, 111]. It is important to note, however, that most of these studies limited the population to either men or women, leaving room for confounding variables. In contrast, consumption exceeding 3–4 drinks/day may or may not increase risk of SCD [104, 105, 111].

Current evidence links heavy alcohol intake with an increased incidence of atrial tachycardias, and AF. In contrast, data regarding other arrhythmias are limited and inconsistent, precluding firm conclusions. Although a few studies suggest that a modest protective association with cardiac arrhythmias may exist with low levels of alcohol consumption, the studies vary in design and power, and likely have various confounding variables. It is difficult to draw conclusions at the time of this review. Randomized controlled trials (RCTs) that study alcohol consumption can pose ethical and logistical challenges, but the potential benefits of definitive causal evidence may justify such efforts if designed ethically. Such evidence would be beneficial for refining clinical guidelines and developing targeted prevention strategies.

## 4. Conclusions

This review highlights current evidence that supports the risks and damages of acute and chronic heavy alcohol on BP, autonomics, CAD, cardiomyopathy, and arrhythmias. While some have suggested that mild to moderate drinking does not pose these harms and may even have beneficial effects, others have suggested the opposite. Mixed findings may be influenced by confounding variables. Further high-quality research is needed to investigate the effects of mild to moderate drinking on the heart. Future research should study specific types of alcohol (wine, beer, spirits, cider) and consider comorbidities that put certain patient populations at higher risk for other acute or chronic cardiac diseases. Other important areas of research on this topic should include a broader population beyond the UK Biobank. Although an extensive database, it is unlikely to be generalizable to other world populations. Further research could target a narrower subset of genetic mutations and their risk on cardiac outcomes together with external factors, or explore even more genetic targets and look for inter-genetic relationships. Other than atrial fibrillation, other arrhythmias deserve more updated conclusions. Finally, additional topics of interest are novel methods to treat alcohol use disorder and potentially preventative treatments against cardiomyopathy in identified high-risk groups.

Given the well-established adverse individual and societal consequences of excessive alcohol consumption, clinical and public health recommendations should emphasize limiting alcohol intake to low levels. Current evidence does not uniformly concur on beneficial effects of low alcohol intake on cardiac health, which should be conveyed to the public. Abstinence from alcohol may be the safest amount of alcohol for the heart.
